# Disconnected – Impaired Interoceptive Accuracy and Its Association With Self-Perception and Cardiac Vagal Tone in Patients With Dissociative Disorder

**DOI:** 10.3389/fpsyg.2018.00897

**Published:** 2018-06-27

**Authors:** Eva Schäflein, Heribert C. Sattel, Olga Pollatos, Martin Sack

**Affiliations:** ^1^Department of Psychosomatic Medicine and Psychotherapy, Klinikum Rechts der Isar, Technical University of Munich, Munich, Germany; ^2^Department of Clinical and Health Psychology, Institute of Psychology and Education, Ulm University, Ulm, Germany

**Keywords:** cardiac vagal tone, dissociation, facial mirror-confrontation, heartbeat detection, interoception, root mean square of successive differences, self-perception

## Abstract

Patients suffering from dissociative disorders are characterized by an avoidance of aversive stimuli. This includes the avoidance of emotions and, in particular, bodily perceptions. In the present pilot study, we explored the potential interoceptive accuracy deficit of patients suffering from dissociative disorders in a heartbeat detection task. Moreover, we investigated the impact of facial mirror-confrontation on interoceptive accuracy and the potential association between cardiac vagal tone derived from heart rate variability and interoceptive accuracy. Eighteen patients suffering from dissociative disorders and 18 healthy controls were assessed with the Mental Tracking Paradigm by Schandry for heartbeat detection at baseline and after confrontations exposing them to their own faces in a mirror (2 min each, accompanied by a negative or positive cognition). During the experiment, cardiac vagal tone was assessed. We used Pearson correlations to calculate potential associations between cardiac vagal tone and interoceptive accuracy. Patients performed significantly worse than the healthy controls in the heartbeat detection task at baseline. They displayed no significant increase in interoceptive accuracy following facial mirror-confrontation. In the patient group, higher cardiac vagal tone was associated with a more precise heartbeat detection performance. Dissociative disorder patients showed a considerable deficit in interoceptive accuracy. Our results fit with the assumption that highly dissociative patients tend to tune out the perceiving of bodily signals. To the extent that bodily signal perception may play a causal role in these disorders, therapeutic approaches enhancing interoceptive accuracy and cardiac vagal tone may be considered important and practicable steps to improve the therapy outcome of this patient group.

## Introduction

Even among experts, there is considerable disagreement about the definition of the concept of dissociation (Holmes et al., 2005; Dell, 2011; Nijenhuis and van der Hart, 2011). There are several diagnostic entities of dissociation. In the following, we present concepts and definitions of dissociation according to the Diagnostic and Statistical Manual of Mental Disorders (DSM) as well as some additional definitions of dissociation.

Dissociative Disorders (DD), as defined in the DSM-5, comprise a ‘disruption of and/or discontinuity in the normal integration of consciousness, memory, identity, emotion, perception, body representation, motor control, and behavior’ (American Psychiatric Association, 2013, p. 291). A Dissociative Disorder Not Otherwise Specified (DDNOS) is present in DD patients who suffer from all of the Dissociative Identity Disorder (DID) criteria except identity alteration or amnesia (American Psychiatric Association, 1994). Even though DDNOS is mentioned as a separate diagnostic category next to DID only in the DSM-IV (American Psychiatric Association, 1994) and not in the DSM-5 (American Psychiatric Association, 2013), it is clinically relevant, as DDNOS is frequent and the diagnostic criteria for ‘full DID’ are tightly defined (Sar, 2011). As the patients of the present investigation are patients that would match with the former diagnosis of DDNOS, we will use the term ‘subthreshold DID’ (‘sDID’) for them throughout the present article. According to the DSM-5 (American Psychiatric Association, 2013), DID is characterized by (A) identity alteration, (B) amnesia, (C) distress about the disorder, (D) the symptoms not corresponding to broadly accepted cultural or religious practice and (E) the symptoms not explained by substance abuse or another medical condition (American Psychiatric Association, 2013).

According to the concept of structural dissociation of the personality (van der Hart et al., 2004), sDID/DID patients have ‘Apparently Normal Part(s) of the Personality’ (responsible for coping with the demands of everyday life) and ‘Emotional Part(s) of the Personality’ (fixated in traumatic memories, which assure survival in situations of severe threat) (van der Hart et al., 2004, 2006). This is reflected psychophysiologically as well: Reinders et al. (2006) have shown that there are considerable differences in psychophysiological reaction (e.g., heart rate, heart rate variability) to a trauma script between ‘neutral identity states’ (i.e., ‘Apparently Normal Parts of the Personality’) that were characterized by a blunted psychophysiological reactivity to the trauma script and ‘traumatic identity states’ (i.e., ‘Emotional Parts of the Personality’) presenting a higher heart rate and lower heart rate variability during the trauma script (Reinders et al., 2006).

Dissociative symptoms are clinically relevant as they are very common in patients with several mental disorders, e.g., in post-traumatic stress disorder (PTSD) and in Borderline Personality Disorder (Sar, 2011) as well as in panic disorder (depersonalization/derealization), obsessive-compulsive disorder (absorption) or depression (depersonalization/derealization) (Soffer-Dudek, 2014). In addition, Levin and Spei (2004) found an association between dissociation and psychopathology in general. A recent meta-analysis by Lyssenko et al. (2017) further supports the notion that dissociative symptoms are prevalent in people with mental disorders.

Clinical experience has demonstrated that patients suffering from DID/sDID avoid self-perception, e.g., during personal hygiene or when seeing their faces in the mirror (see also Frewen et al., 2011). In particular, those patients tune out the perception of emotions and especially of bodily signals (van der Hart et al., 2006; Boon et al., 2011). According to Hayes et al. (2004), dissociation can be related to the construct of ‘experiential avoidance,’ a construct that is defined as ‘the phenomenon that occurs when a person is unwilling to remain in contact with particular private experiences (e.g., bodily sensations, emotions, thoughts, memories, and behavioral predispositions) and takes steps to alter the form or frequency of these events and the context that occasion them’ (Hayes et al., 1996, p. 1154). Dyer et al. (2015) found associations between specific areas of the body which were associated with trauma and highly aversive emotions in patients suffering from post-traumatic stress disorder. That is why the authors conclude that the perception of the patient’s body might trigger traumatic memories (Dyer et al., 2015).

A construct describing the afferent information from the viscera, e.g., from the heart, is interoception (Herbert and Pollatos, 2008; Garfinkel et al., 2015). Craig (2003, p. 500) defines interoception as ‘the sense of the physiological condition of the body,’ e.g., hunger and thirst. Hoeschel et al. (2008) have shown that dissociative symptom severity is correlated to a compromised fluid intake, a fact that might be linked to impaired interoception in highly dissociative individuals. Numerous studies have investigated interoception in various mental disorders (see Schulz and Vögele, 2015, for a review). For instance, patients with panic disorders and anxiety disorders in general have shown heightened interoceptive accuracy (Domschke et al., 2010). In contrast, there is evidence for compromised interoceptive accuracy in patients suffering from personality disorders (Mussgay et al., 1999), conversion disorders (Ricciardi et al., 2016), or eating disorders (Pollatos et al., 2008; Herbert and Pollatos, 2014). Patients with Depersonalization-Derealization Disorder (Michal et al., 2014) or with Borderline Personality Disorder (Hart et al., 2013) did not exhibit compromised interoceptive accuracy when compared to healthy controls, contrary to the authors’ hypotheses.

According to James (1884), bodily perceptions are crucial for the experience of emotion and precede the emotional experience. Experts distinguish between ‘interoceptive accuracy’ (IAc) meaning the accuracy of the perception of interoceptive signals that can be examined using heartbeat perception performance (also called ‘cardiosensibility’) and ‘interoceptive sensibility’ as the self-evaluation of subjective interoception (assessed by interviews or questionnaires) (Garfinkel et al., 2015). The ‘Multidimensional Assessment of Interoceptive Awareness’ (MAIA, Mehling et al., 2012), for instance, is a questionnaire that assesses interoceptive sensibility on eight subscales (‘Noticing,’ ‘Not-Distracting,’ ‘Not-Worrying,’ ‘Attention Regulation,’ ‘Emotional Awareness,’ ‘Self-Regulation,’ ‘Body Listening,’ and ‘Trusting’) using items such as ‘I listen for information from my body about my emotional state’ or ‘I feel my body is a safe place’.

Self-perception and cardiac vagal tone have proven to be variables associated with IAc. In healthy participants, Weisz et al. (1988) and Ainley et al. (2012) have reported an improvement in interoception in the course of self-perception of participants’ faces in a mirror, and Ainley et al. (2013) have further shown this IAc-improving effect for a paradigm including bodily as well as narrative self-aspects. Interestingly, Pollatos et al. (2016) found that in Anorexia Nervosa patients, interoceptive accuracy was higher when focusing on another person’s face than when being confronted with their own faces, whereas the opposite was the case for healthy controls. We have recently shown altered self-perception in the sDID patient sample of the present investigation: mirror-confrontation with their own faces constituted a serious stressor for them regarding self-reported stress and state dissociation, whereas healthy controls did not exhibit these stress reactions (Schäflein et al., 2016, 2018). The effect of facial mirror-confrontation on IAc has not been experimentally reproduced in sDID/DID patients. Recent evidence has shown that, besides self-perception, cardiac vagal tone is linked to IAc (Pollatos et al., 2014). In healthy participants, Pollatos et al. (2014) have shown a correlation between higher IAc and higher vagal control of the heart (*r* = 0.48, *p* < 0.05). Thus far, there is no research replicating this finding in a clinical sample and especially not in sDID/DID patients.

Previous research has shown a correlation between dissociative symptoms and a poor psychotherapy outcome (Michelson et al., 1998; Rufer et al., 2006; Spitzer et al., 2007; Kleindienst et al., 2011). Angelovski et al. (2016) describe an association between a higher baseline heart rate variability and higher heart rate variability reactivity with a better clinical outcome for a psychotherapeutic intervention in patients with pain dominant somatoform disorders. The authors speculate that this might be linked to self-regulation and emotional learning capacities (Angelovski et al., 2016). Research on the potential reasons for the poor therapeutic outcome of highly dissociative patients is urgently needed (Lanius, 2015). There is ample evidence that interoception is correlated to emotional experience (Schandry, 1981; Wiens, 2005; Herbert and Pollatos, 2008, 2012; Füstös et al., 2012; Terasawa et al., 2013). Ebner-Priemer et al. (2009) have demonstrated that compromised emotional learning is associated with a high level of state dissociative experiences in Borderline Personality Disorder and speculate that a lack of emotional engagement might be responsible for the poor therapy outcome of highly dissociative patients. Another study by Jaycox et al. (1998) has shown that, besides habituation, emotional engagement is crucial for a good therapy outcome in PTSD exposure therapy.

We were interested to find out if IAc was impaired in sDID patients, a potential pathomechanism that might be associated with poor emotional engagement and thus a poor psychotherapy outcome in sDID/DID patients. Furthermore, we aimed to investigate whether the IAc of sDID patients might improve following facial mirror-confrontation. Moreover, it was of interest to us to see whether or not cardiac vagal tone derived from heart rate variability was linked to interoception in sDID patients.

We thus hypothesized a significant difference in baseline IAc (heartbeat detection score) between DD patients and healthy controls (HCs) (hypothesis 1). Furthermore, we assumed a significant increase in IAc in both patients (hypothesis 2a) and HCs (hypothesis 2b) when exposing them to their faces in a mirror. Additionally, we expected a significant association between cardiac vagal tone derived from heart rate variability and IAc in both patients (hypothesis 3a) and HCs (hypothesis 3b).

## Materials and Methods

### Participants

The local ethics committee approved the investigation (proposal 1/14 S) in accordance with the Helsinki Declaration. All participants provided their written informed consent. The present study is a pilot study for the estimation of observed effect sizes. The results of the current study are based on the same sample as the findings of Schäflein et al. (2016) and of Schäflein et al. (2018). We sent invitations to 60 patients. Twenty-two of them did not reply or were not interested in participating. Eighteen patients did not meet the inclusion criteria. Inclusion and exclusion criteria will be specified below. Another two patients were not able to follow the instructions during the experiment. They repeatedly closed their eyes during the experiment and faded out due to severe and continuous detachment symptoms. Two hundred HCs replied to our notices and advertisements in the hospital and on the intranet. The two groups were matched for gender, age and body mass index, parameters that are known to influence psychophysiological measures. We chose 23 HC candidates that were suitable according to the matching criteria (age, gender, and body mass index) for matching. Four HC candidates were excluded because they suffered from a current mental disorder, and one of them was not able to participate due to language problems.

Exclusion criteria for all participants were a severe internal or neurological disorder or the taking of beta blockers or benzodiazepines. Patients with a current severe depressive episode, lifetime psychotic disorder or lifetime substance abuse were excluded. The inclusion criterion for patients was a score of 10 or more points on the Mini-SCID-D interview (Gast et al., 1999). We excluded HCs with a current mental disorder.

Eighteen (17 female) patients with sDID and 18 HCs (17 female) participated in the study. Patients were consecutive inpatients or outpatients at a center specialized in psychotraumatology at the Department of Psychosomatic Medicine and Psychotherapy, Klinikum rechts der Isar, Technical University of Munich, Germany. HCs were employees and medical students at Klinikum rechts der Isar, Technical University of Munich. Sociodemographic data and sample characteristics are shown in **Table 1**.

**Table 1:** Sociodemographic data and sample characteristics.

	sDID	HCs	Group comparison
			
	*N* = 18	*N* = 18	*p*
Age (*M*; *SD*)	41.7 (8.3)	41.1 (10.0)	0.86
Education (% high-school diploma)	50.0	61.1	0.50
Gender (% female)	94.4	94.4	1.00
BMI (*M*; *SD*)	23.6 (4.1)	24.7 (2.9)	0.40

*Between-group differences: t-tests for independent samples (continuous data)*/χ*^2−^tests (nominal data). sDID, patients with subthreshold Dissociative Identity Disorder; HCs, healthy controls; M, mean; SD, standard deviation; BMI, body mass index.*

There were no relevant differences in age, gender, percentage of high school diploma holders, or body mass index between the two groups (**Table 1**). Patients on average had had 5.1 (*SD* = 5.0) months of lifetime psychosomatic and 2.3 (*SD* = 4.0) months of lifetime psychiatric inpatient treatment. All but one patient had had outpatient psychotherapy treatment. Comorbidities were frequent and included major depression (*n* = 9, 50%) or major depression combined with an anxiety, obsessive-compulsive or eating disorder (*n* = 6, 33.3%). 44.4% of the sDID patients took antidepressant or neuroleptic drugs, and 27.8% of them took additional drugs other than psychopharmaceuticals. Only one of the HCs had had outpatient psychotherapy treatment. None of the HCs took psychopharmaceuticals, and 16.7% of them took additional drugs other than psychopharmaceuticals.

### Instruments

We used the validated German translations of all instruments employed in this study.

#### Interviews

Subthreshold DID and PTSD diagnoses were assessed using the Mini-SCID-D interview (Gast et al., 1999; Steinberg et al., 1992, unpublished), which is the short form of the SCID-D interview (Steinberg et al., 1992, unpublished) and the SCID-PTSD interview (Wittchen et al., 1997; First et al., 2012) according to DSM-IV. The Mini-SCID-D and the SCID-D interview (Steinberg, 1994; Gast et al., 1999) consist of the subscales amnesia, depersonalization, derealization, identity disturbance, and identity alteration (Steinberg, 1994; Gast et al., 1999) and thus comprise fragmentation and detachment symptoms.

#### Self-Report Measures

Trait dissociation was assessed with the Dissociative Experiences Scale (DES, Bernstein and Putnam, 1986). The Impact of Event Scale (IES, Horowitz et al., 1979) was administered to measure the severity of trauma-related symptoms. We quantified child abuse and neglect using the Childhood Trauma Questionnaire CTQ and its five subscales (emotional abuse, physical abuse, sexual abuse, emotional neglect, and physical neglect) (Bernstein and Fink, 1998). The Multidimensional Assessment of Interoceptive Awareness (MAIA) (Mehling et al., 2012) is a 32-item questionnaire consisting of eight subscales (‘Noticing,’ ‘Not-Distracting,’ ‘Not-Worrying,’ ‘Attention Regulation,’ ‘Emotional Awareness,’ ‘Self-Regulation,’ ‘Body Listening,’ ‘Trusting’). Items are categorized on a five point Likert scale from ‘0’ = ‘never’ to ‘5’ = ‘always’. The MAIA total score is the mean of all items, some of them inversely coded. A higher MAIA score signifies better interoceptive sensibility. The Acceptance and Action Questionnaire (AAQ, Hayes et al., 2004) was used to measure experiential avoidance. We used the Subjective Units of Disturbance (SUD) Scale to quantify subjective distress on a scale of 0 (‘no distress’) to 10 (‘maximum distress’) (Wolpe, 1969).

### Procedures

**FIGURE 1:**
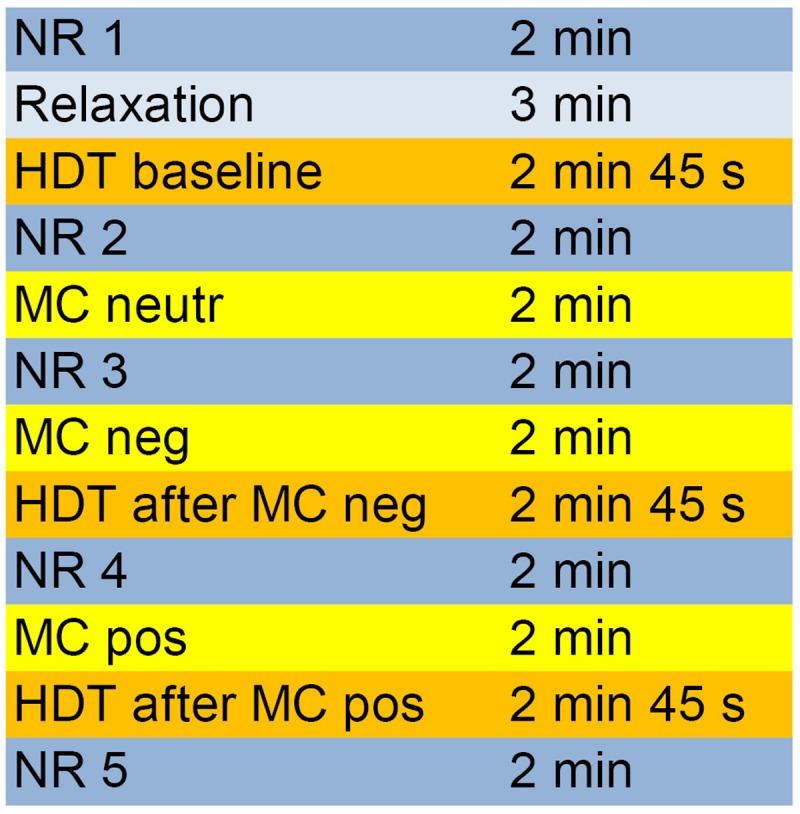
Course of the different measurement phases of the experiment. HDT, heartbeat detection task; MC, mirror-confrontation; NR, neutral reference (imagining washing the dishes); neutr, neutral (no cognitive accompaniment); neg, negative (with negative cognitive accompaniment); pos, positive (with positive cognitive accompaniment).

The entire experimental process and the different periods are depicted in **Figure 1**.

Participants were seated in a comfortable chair 1 m in front of a mirror (40 cm × 40 cm) that reflected their faces. The experiment comprised three mirror-confrontation phases (2 min each, **Figure 1**) during which participants were instructed to either look just at their faces or to silently think about a negative and then a positive cognition during facial mirror-confrontation. We assessed IAc (see section ‘Heartbeat Detection Task’) using heartbeat detection tasks at three points, at baseline, directly after facial mirror-confrontation accompanied by a negative cognition (2 min duration) and directly following facial mirror-confrontation accompanied by a positive cognition (2 min duration) (**Figure 1**). The mirror was covered during the whole experimental procedure (including the three heartbeat detection tasks, see section ‘Heartbeat Detection Task’) except for the mirror-confrontation phases.

Participants were asked to choose individual, pre-defined negative and positive cognitions about themselves from the Eye Movement Desensitization and Reprocessing Manual (Shapiro, 2001). They should report their most disturbing negative cognition which had to induce a Subjective Units of Disturbance (SUD, Wolpe, 1969) score of at least 7 to be eligible as an accompanying cognition. In HCs, a SUD score of 7 or more was not present since HCs reported they could not think about themselves in such a negative way. Before, between and after the mirror-confrontation periods and the heartbeat detection tasks, neutral reference conditions (2 min each, sitting and imagining washing the dishes) were performed (**Figure 1**). Before the baseline IAc measurement, participants performed a relaxation task (Wengenroth, 2012, p. 143) (adapted version) to limit anticipatory anxiety and for ethical reasons. During the whole experiment, electrocardiography (ECG) and impedance cardiography (ICG) data were collected continuously. For ethical reasons (mirror-confrontation with positive cognition should be at the end of the experiment), we did not permute the mirror conditions.

### Heartbeat Detection Task

The Mental Tracking Task by Schandry (1981) served to assess heartbeat detection accuracy. The task is well-validated, reliable and widely used (Knoll and Hodapp, 1992; Mussgay et al., 1999; Pollatos et al., 2007). Participants were asked to mentally track (i.e., to count silently) their heartbeats in three time intervals of 25, 35, and 45 s without taking their pulse or facilitating heartbeat detection in any other way. Between these time intervals, there were short breaks of 30 s. Start and stop signals for each counting phase were given by the experimenter (ES). After each time interval, participants wrote down the number of heartbeats detected. As we asked participants to perform the heartbeat detection task three times, we permuted the order of the three time intervals in a balanced design.

**FIGURE 2:**
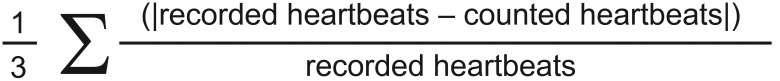
Formula used to calculate the heartbeat detection score.

During the Mental Tracking Task, we monitored heart rates by means of an ECG measured by the Vrije Universiteit Ambulatory Monitoring System (VU-AMS, Vrije Universiteit, Department of Psychophysiology, Amsterdam, Netherlands) Model 5 FS [see section ‘Impedance Cardiography and Vrije Universiteit Ambulatory Monitoring System (VU-AMS)’]. The Mental Tracking Task requires intervals of 25, 35, and 45 s. During these time periods, we assessed cardiac vagal tone [see section ‘Impedance Cardiography and Vrije Universiteit Ambulatory Monitoring System (VU-AMS)’] and calculated a mean cardiac vagal tone score for each heartbeat detection task. For each of the three heartbeat detection tasks, we calculated a heartbeat detection score (HBDS) as the mean of the three intervals using the formula shown in **Figure 2**.

Using this transformation, the HBDS can vary between 0 and 1. Higher scores indicate smaller differences between recorded and perceived heartbeats and thus higher IAc.

### Impedance Cardiography and Vrije Universiteit Ambulatory Monitoring System (VU-AMS)

The validated Vrije Universiteit Ambulatory Monitoring System (VU-AMS, Vrije Universiteit, Department of Psychophysiology, Amsterdam, Netherlands) served to assess peripheral psychophysiological activity by recording ECG and ICG using a sample rate of 1000 Hz (de Geus et al., 1995; de Geus and van Doornen, 1996; Willemsen et al., 1996; Riese et al., 2003). We used the Root Mean Square of Successive Differences (RMSSD) as a vagal index derived from heart rate variability to assess cardiac vagal tone (Malik et al., 1996). We employed the natural logarithm of RMSSD, lnRMSSD, for further analysis because of the skewed distribution of the RMSSD (Malik et al., 1996). An increase in lnRMSSD means an increase in cardiac vagal tone. We processed physiological data using the VU-DAMS 3.2 software. Suspicious beats were detected automatically by the VU-AMS. We visually inspected and manually corrected or deleted suspicious beats and artifacts using the procedures in the Vrije Universiteit Data Analysis and Management Software (VU-DAMS) manual (VU-DAMS, 2013). All files were processed.

### Statistical Analyses

Statistical analyses were conducted using SPSS version 22.0, applying a statistical threshold for probability of *p* < 0.05 (two-sided). For group comparisons, *t*-tests for independent samples were computed. In the case of nominal data, χ^2^ tests were used. To analyze data collected at different time points (within-group differences), we computed linear mixed models with the participant as a random effect and time and group as fixed effects (Singer, 1998). To assess the association between cardiac vagal tone and IAc, we used parametric correlations. We calculated between-group differences of subsequent experimental conditions using ANOVAs and controlled for the initial value of the dependent variable, respectively.

## Results

### Psychometric Sample Characteristics

**Table 2** shows the psychometric data of the sDID patients and the HCs. Mini-SCID-D interviewing to determine the severity of dissociative symptoms resulted in a score of 11.39 (*SD* = 1.09, range 5–15) points for the sDID group, indicating that all patients suffered from detachment symptoms (depersonalization/derealization) and, additionally, from compartmentalization symptoms (amnesia/identity disturbance/identity alteration). All of the patients had sDID (all DID criteria but amnesia or but identity alteration), but not a full DID. All patients met the criteria for a comorbid PTSD according to SCID-PTSD, but, as clinical expert interviews revealed, sDID was the main diagnosis and main complaint in every patient tested. All patients reported multiple traumatization (*M* = 3.2 traumatizations, *SD* = 1.3). In contrast to the HCs, sDID patients exhibited more intense dissociative symptoms in the DES, severe PTSD symptomatology in the IES, reported considerably high childhood abuse and neglect intensity in the CTQ, remarkably compromised interoceptive sensibility in the MAIA and notably high experiential avoidance in the AAQ. The dissociation score according to the Mini-SCID-D interview was zero for each of the HCs. HCs comprised both traumatized and non-traumatized individuals. However, their average childhood traumatization scores were significantly lower than those of the patients. Their overall psychopathological symptoms were considerably lower than those of the patients (**Table 2**).

**Table 2:** Psychometric data.

		sDID	HCs	Group comparison
	Questionnaire (abbreviation) (range)	*M* (*SD*)	*M* (*SD*)	*p*
Dissociation	Mini-SCID-D total score (0–15)	11.39 (1.09)	0.00 (0.00)	<0.001^∗^
	Mini-SCID-D amnesia (0–3)	2.44 (0.98)	0.00 (0.00)	<0.001^∗^
	Mini-SCID-D depersonalization (0–3)	3.00 (0.00)	0.00 (0.00)	<0.001^∗^
	Mini-SCID-D derealization (0–3)	2.11 (108)	0.00 (0.00)	<0.001^∗^
	Mini-SCID-D identity disturbance (0–3)	2.33 (0.49)	0.00 (0.00)	<0.001^∗^
	Mini-SCID-D identity alteration (0–3)	1.50 (0.92)	0.00 (0.00)	<0.001^∗^
	Dissociative Experiences Scale (DES) (0–100%)	27.86 (9.28)	4.35 (2.79)	<0.001^∗^
Trauma	PTSD (diagnosed with SCID-PTSD interview)	Yes	No	
	Impact of Event Scale (IES) (0–75)	54.28 (11.85)	–	–
	Childhood Trauma Questionnaire (CTQ) (25–125)	82.50 (15.75)	29.25 (3.25)	<0.001^∗^
	CTQ emotional abuse (5–25)	18.90 (4.85)	6.20 (1.15)	<0.001^∗^
	CTQ physical abuse (5–25)	12.90 (4.70)	5.15 (0.40)	<0.001^∗^
	CTQ sexual abuse (5–25)	16.45 (5.60)	5.00 (0.00)	<0.001^∗^
	CTQ emotional neglect (5–25)	21.45 (2.30)	7.55 (2.00)	<0.001^∗^
	CTQ physical neglect (5–25)	12.80 (3.80)	5.30 (0.70)	<0.001^∗^
Interoceptive sensibility	Multidimensional Assessment of Interoceptive Awareness (MAIA) (0–5)	1.70 (0.66)	3.25 (0.56)	<0.001^∗^
Experiential avoidance	Acceptance and Action Questionnaire (AAQ) (9–63)	45.11 (6.72)	22.22 (3.46)	<0.001^∗^

*Between-group differences: t-tests for independent samples. sDID, patients with subthreshold Dissociative Identity Disorder; HCs, healthy controls; M, mean; SD, standard deviation; SCID-D, Structural Clinical Interview for DSM-IV Dissociative Disorders; PTSD, post-traumatic stress disorder; DES, Dissociative Experiences Scale; IES, Impact of Event Scale; CTQ, Childhood Trauma Questionnaire; MAIA, Multidimensional Assessment of Interoceptive Awareness; AAQ, Acceptance and Action Questionnaire;*^∗^*p < 0.05.*

### Interoceptive Accuracy Results at Baseline and in the Course of the Facial Mirror-Confrontation Paradigm

**FIGURE 3:**
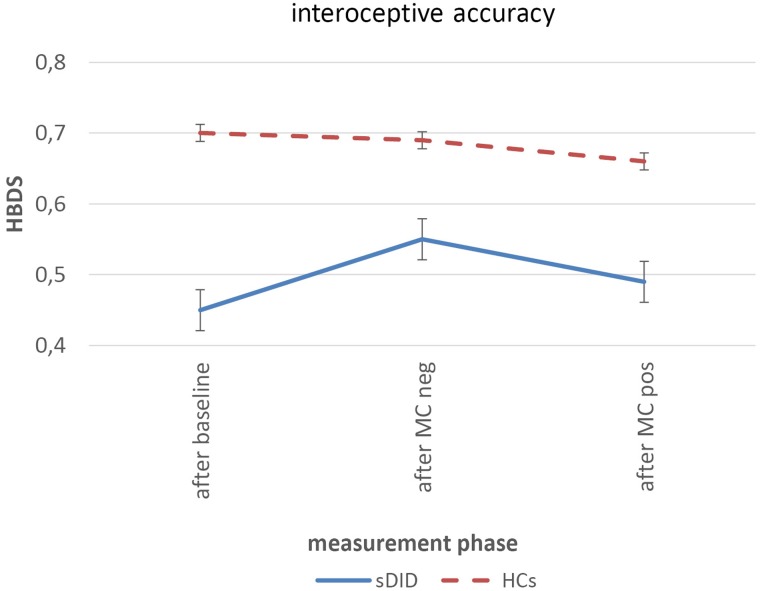
Heartbeat detection score over the course of the experiment. Within-group differences: linear mixed models, between-group differences: ANOVAs controlling for initial value of HBDS. sDID, patients with subthreshold Dissociative Identity Disorder; HCs, healthy controls; MC, mirror-confrontation; neg, negative (negative cognitive accompaniment); pos, positive (positive cognitive accompaniment); HBDS, heartbeat detection score.

**Figure 3** shows the courses of the heartbeat detection score for sDID patients (blue) and HCs (red) at the three measurement points: at the baseline assessment, after the facial mirror-confrontation with negative cognitive accompaniment and after the facial mirror-confrontation with positive cognitive accompaniment.

**Table 3** depicts the baseline heartbeat detection scores (HBDS) of patients and HCs, the mean HBDS values and standard errors for each measurement period, the within-group changes in HBDS for the sDID patients and for the HCs in the course of the experiment and the between-group differences in the course of the experiment when controlling for the initial HBDS value. There was a significant difference in the HBDS between the sDID patients and the HCs at baseline [*T*(df) = *T*(28.0) = −2.776; *p* = 0.010]. For both groups, there were no significant changes from the HBDS at baseline to the HBDS after the facial mirror-confrontation with negative cognitive accompaniment [sDID: ES (CI) = 0.30 (−0.96 to 0.36), *p* = 0.14; HCs: ES (CI) = 0.05 (−0.60 to 0.70), *p* = 0.77]. Similarly, there were no significant changes going from the HBDS after the facial mirror-confrontation with negative cognitive accompaniment to the HBDS after the facial mirror-confrontation with positive cognitive accompaniment in both groups [sDID: ES (CI) = 0.18 (−0.48 to 0.83), *p* = 0.14; HCs: ES (CI) = 0.15 (−0.50 to 0.81), *p* = 0.21]. Controlling for the initial values of the HBDS, ANOVAs did not yield any significant between-group differences between sDID patients and HCs neither for the HBDS after the facial mirror-confrontation accompanied by the negative cognition (*F* = 0.13, *p* = 0.72) nor for the HBDS after the facial mirror-confrontation accompanied by the positive cognition (*F* = 0.16, *p* = 0.70) (**Table 3**).

**Table 3:** Within-group courses of the heartbeat detection scores for sDID patients and HCs (left) and between-group differences calculated using ANOVAs, controlled for initial heartbeat detection score value (right).

	sDID				HCs				sDID compared to HCs^2^	
	*M* (SE)	*F*	ES (95% CI)	*p*^1^	*M* (*SE*)	*F*	ES (95% CI)	*p*^1^	*F*	*p*
HBDS at baseline	0.45 (0.03)				0.70 (0.09)					
HBDS after MC neg	0.55 (0.02)	2.36	0.30 (−0.96 to 0.36)	0.14	0.69 (0.08)	0.09	0.05 (−0.60 to 0.70)	0.77	0.13	0.72
HBDS after MC pos	0.49 (0.03)	2.45	0.18 (−0.48 to 0.83)	0.14	0.66 (0.09)	1.69	0.18 (−0.50 to 0.81)	0.21	0.16	0.70

*^1^Mean within-group difference related to precedent measuring period calculated by linear mixed models; ^2^between-group differences: ANOVAs controlling for initial value of the dependent variable. CI, confidence interval; sDID, patients with subthreshold Dissociative Identity Disorder; HCs, healthy controls; M, mean; SE, standard error; HBDS, heartbeat detection score; MC, mirror-confrontation; neg, negative (with negative cognitive accompaniment); pos, positive (with positive cognitive accompaniment); ES, effect size.*

### Correlations Between Cardiac Vagal Tone and Interoceptive Accuracy

**Table 4** depicts Pearson correlations between cardiac vagal tone (lnRMSSD) and IAc in the heartbeat detection tasks for sDID patients and healthy controls. In sDID patients, there were significant associations with high effect sizes at baseline and after mirror-confrontation with positive cognitive accompaniment (baseline: *r* = 0.55, *p* = 0.02; after mirror-confrontation with positive cognitive accompaniment: *r* = 0.54, *p* = 0.02). In the HCs, there were no substantial associations between cardiac vagal tone and IAc (baseline: *r* = −0.08, *p* = 0.74; after mirror-confrontation with negative cognitive accompaniment: *r* = −0.25, *p* = 0.33; after mirror-confrontation with positive cognitive accompaniment: *r* = −0.29, *p* = 0.25) (**Table 4**).

**Table 4:** Correlation between cardiac vagal tone (lnRMSSD) and interoceptive accuracy (heartbeat detection score).

	sDID		HCs	
	*r*	*p*	*r*	*p*
HBDS at baseline	0.55	0.02^∗^	−0.08	0.74
HBDS after MC neg	0.33	0.19	−0.25	0.33
HBDS after MC pos	0.54	0.02^∗^	−0.29	0.25

*ln, natural logarithm; RMSSD, root mean square of successive differences; sDID, patients with subthreshold Dissociative Identity Disorder; HCs, healthy controls; HBDS, heartbeat detection score; MC, mirror-confrontation; neg, negative (with negative cognitive accompaniment); pos, positive (with positive cognitive accompaniment); p < 0.05.*

## Discussion

We compared IAc assessed by a heartbeat detection task between sDID patients suffering from both detachment and compartmentalization symptoms and HCs at baseline and in the course of a facial mirror-confrontation paradigm. Furthermore, we investigated potential correlations between IAc and cardiac vagal tone. In comparison to the HCs, we observed an IAc deficit in sDID patients at baseline. There was no substantial increase in IAc following facial mirror-confrontation for both patients and HCs. In sDID patients, higher cardiac vagal tone was correlated to a higher heartbeat detection accuracy.

In accordance with hypothesis 1, we found a significant IAc deficit in sDID patients compared to the HCs. Our outcome showing significantly attenuated IAc in sDID patients compared to HCs is in line with results from previous studies demonstrating IAc deficits in patients affected by personality disorders (Mussgay et al., 1999) or conversion disorders (Ricciardi et al., 2016). Moreover, a case study by Sedeño et al. (2014) showing an IAc deficit in a Depersonalization-Derealization Disorder patient compared to HCs fits with our results. Apart from this, results by Schaefer et al. (2012) reporting a correlation between lower IAc and higher symptom severity in somatoform disorder patients are also in accordance with our findings, as our sample showed severe general psychopathology (see section ‘Psychometric Sample Characteristics,’ **Table 2**).

In contrast, our results are not in line with research failing to show an IAc deficit in patients with Borderline Personality Disorder (Hart et al., 2013) or with Depersonalization-Derealization Disorder (Michal et al., 2014) compared to HCs. Dissociative symptoms are a diagnostic criterion for Borderline Personality Disorder (American Psychiatric Association, 2013). It is thus possible that including dissociative symptoms as a covariate in the study by Hart et al. (2013) might have shown an IAc deficit depending on dissociative symptom severity. Furthermore, our findings are not in line with the observation that patients suffering from panic disorder who often display depersonalization/derealization (Soffer-Dudek, 2014) have heightened IAc (Domschke et al., 2010). Compared to their findings and to the results of Michal et al. (2014), our data might be interpreted as related to fragmentation/compartmentalization symptoms such as amnesia or identity alteration that were present in our patients in addition to detachment symptoms (Holmes et al., 2005). When interpreted in the light of the theory of structural dissociation of the personality (van der Hart et al., 2004, 2006), the role of fragmentation symptoms in the present study might imply that our patients have avoided their ‘Emotional Parts of the Personality,’ which are linked to trauma-associated emotions and thus most probably also to bodily signals. They might rather have been in their ‘Apparently Normal Part of the Personality,’ the personality state responsible for the demands of everyday life and detached from emotional experience linked to traumatic memories. Therefore, they might also have been detached from correspondent bodily signals, given the well-documented link between emotional experience and interoception/perception of signals arising from the body (Schandry, 1981; Wiens, 2005; Herbert and Pollatos, 2008, 2012; Füstös et al., 2012; Terasawa et al., 2013).

Contrary to hypotheses 2a and 2b, we did not observe a significant increase in IAc following facial mirror-confrontation in either sDID patients or HCs. Looking at the effect size, one may speculate that with a larger sample the small increase from the heartbeat detection task at baseline to the heartbeat detection task after the mirror-confrontation with negative cognitive accompaniment might have reached statistical significance in our patient sample. Our finding of no significant increase in IAc following facial mirror-confrontation in both sDID patients and HCs contradicts previous findings by Ainley et al. (2012). Those authors found a significant increase in IAc linked to facial mirror-confrontation in healthy participants. Since mirror-confrontation with their faces has proven to be associated with stress and state dissociation in the sDID patient sample of the present investigation (Schäflein et al., 2016, 2018), increased self-focus by facial mirror-confrontation might have resulted in an increase in IAc, whereas the simultaneous stress and state dissociation experience might have diminished IAc. Fairclough and Goodwin (2007), for instance, have shown an IAc decrease associated with stress in healthy women. Additionally, participants in our study underwent the heartbeat detection tasks following and not during facial mirror-confrontation. The effects of facial mirror-confrontation might already have diminished as the participants had executed the heartbeat detection tasks after the facial mirror-confrontations. Another potential reason why we were not able to detect the hypothesized IAc increase might be that sDID patients might have avoided self-perception. This interpretation is in line with Pollatos et al. (2016) demonstrating that IAc was enhanced when looking at another person’s face and decreased when looking at their own faces in a sample of anorexia nervosa patients. The authors conclude that confronting anorexia nervosa patients with their faces might lead to increased body-related avoidance and thus a decrease in IAc (Pollatos et al., 2016).

In the HCs, there was also no significant increase in IAc associated with mirror-confrontation. At first sight, this finding contradicts three aforementioned investigations reporting a significant increase in IAc during mirror-confrontation with one’s face (Weisz et al., 1988; Ainley et al., 2012) and during a task leading attention toward bodily or narrative self-aspects (Ainley et al., 2013) in healthy participants. Our finding concerning the HCs furthermore seems to be inconsistent with work by Pollatos et al. (2016) demonstrating an increase in IAc going along with explicit self-focus in healthy study participants. Again, a potential explanation might be that we performed the heartbeat detection tasks after and not during the mirror-confrontation intervals. Furthermore, the HCs exhibited high baseline IAc. The lack of an increase in IAc after HCs were exposed to their faces in the mirror might thus be associated with a ceiling effect.

In keeping with hypothesis 3a, we observed significant positive correlations between cardiac vagal tone and IAc at baseline and after mirror-confrontation accompanied by the positive cognition in sDID patients. There was no such correlation after mirror-confrontation accompanied by a negative cognition. Our results suggest that patients with a higher cardiac vagal tone detect a greater proportion of their heartbeats and that this effect might be attenuated by retrieving a negative cognition, i.e., that the negative cognition possibly leads to a decoupling of the association between cardiac vagal tone and interoceptive accuracy. The finding of a significant positive correlation between cardiac vagal tone and IAc in sDID patients is in line with Pollatos et al. (2014) reporting on an association between higher vagal control of the heart and higher IAc in healthy participants. To our knowledge, our study is the first to replicate this finding in a clinical population and especially in sDID patients. Cardiac vagal tone might be an important factor contributing to psychotherapy outcome in sDID patients as well, as shown by Angelovski et al. (2016) for patients with pain dominant somatoform disorders.

Herein, we show for the first time a link between cardiac vagal tone and IAc, a construct linked to emotional experience (Schandry, 1981; Wiens, 2005; Herbert and Pollatos, 2008, 2012; Füstös et al., 2012; Terasawa et al., 2013), in sDID patients. Evidence suggesting an association of impaired emotional experience with a poor psychotherapy outcome in post-traumatic disorders (Jaycox et al., 1998) and highly dissociative individuals (Ebner-Priemer et al., 2009) underlines that our findings might make an important contribution toward understanding the pathophysiology of poor emotional learning and thus compromised psychotherapy outcome in patients suffering from severe dissociative symptoms. When considering the pivotal role of interoception for emotional experience (Schandry, 1981; Wiens, 2005; Herbert and Pollatos, 2008, 2012; Füstös et al., 2012; Terasawa et al., 2013) and the herein-found link between cardiac vagal tone and interoception, one might speculate that deficiencies of both cardiac vagal tone and interoception might be psychophysiological correlates of poor emotional experience and thus might be pathways contributing to the thus far poor psychotherapy outcome of sDID/DID patients. The psychotherapy outcome of this severely disabled patient group might be improved if psychotherapeutic techniques focusing on the perception of the own body and bodily signals and aiming at heightening cardiac vagal tone were included into their psychotherapy.

In contrast to hypothesis 3b, we were not able to detect significant correlations between cardiac vagal tone and IAc in the HCs. This is most likely due to their relatively high baseline heartbeat detection score. Our HCs were mostly hospital employees and medical students, who probably have a better idea of a normal heart rate than the average population. Considering a ceiling effect, it is thus difficult to detect variables enhancing a high baseline heartbeat detection ability. Thus, our findings might not automatically contradict previous results by Pollatos et al. (2014) describing a positive correlation between vagal tone and interoceptive accuracy in a healthy population.

Our finding of impaired baseline IAc and of low interoceptive sensibility assessed by the MAIA questionnaire in sDID patients might be interpreted as fitting with the theory of experiential avoidance (Hayes et al., 2004). This theory implies that it is difficult for people high in experiential avoidance to ‘remain in contact with particular private experiences (e.g., bodily sensations, emotions, […])’ (Hayes et al., 1996, p. 1154). Psychometrically, our sDID patient sample exhibited pronounced experiential avoidance in the Acceptance and Action Questionnaire (**Table 2**). However, it is not possible to conclude from our data whether impaired IAc is owed to avoidance, to inability, or both. Further studies including the subjective aversiveness of the heartbeat detection tasks could shed more light on this question. Considering data by Dyer et al. (2015) assuming the own body is a trigger in post-traumatic conditions, impaired IAc in our patients might be due to avoiding getting in touch with the trauma-associated own body.

Our data support the notion of the definition of dissociation, suggesting that a disruption in the normal integration of emotion, perception and body representation might be associated with impaired IAc in sDID patients. Given the differences between ‘Apparently Normal Parts of the Personality’ and ‘Emotional Parts of the Personality’ in DID patients concerning their reaction to a trauma script (Reinders et al., 2006), our patients might have been in their ‘Apparently Normal Part of the Personality’ state when performing the baseline heartbeat detection task. Avoidance of bodily signals and impaired IAc might thus be ‘dissociative part’-dependent. Consequently, it would be interesting to replicate the experiment with DID patients in their ‘Apparently Normal Part of the Personality’ and ‘Emotional Part of the Personality’ states in order to differentiate systematically between these two conditions.

Considering our findings, it is possible that a good ability to regulate cardiac vagal tone might help sDID/DID patients to overcome their tuning-out of the perception of emotions and especially of bodily perceptions (van der Hart et al., 2006; Boon et al., 2011). Enhancing cardiac vagal tone and interoceptive abilities might thus counteract dissociation-specific feelings of disconnection from their bodies in sDID/DID. According to our findings, one might speculate that enhancing bodily perception, e.g., using body therapy or mindfulness exercises, as well as monitoring and influencing cardiac vagal tone, e.g., by biofeedback, might constitute psychotherapy targets for enhancing IAc in sDID/DID patients. Given the importance of bodily signals in general (e.g., James, 1884) and of interoceptive information in particular (Schandry, 1981; Wiens, 2005; Herbert and Pollatos, 2008, 2012; Füstös et al., 2012; Terasawa et al., 2013) for the experience of emotion, we suggest that impaired IAc might be a crucial factor associated with impaired emotional experience and thus emotional learning that most probably also occurs during psychotherapy in highly dissociative patients (Ebner-Priemer et al., 2009). This again might contribute to the poor psychotherapy outcome linked to dissociation described in previous research (Michelson et al., 1998; Rufer et al., 2006; Spitzer et al., 2007; Kleindienst et al., 2011, 2016). Another clinical implication of our results might be that impaired interoception most probably might be an explanation for clinically reported compromised fluid intake maintaining dissociation as reported by Hoeschel et al. (2008).

### Limitations and Future Directions

Our study has several limitations. Due to the pilot character of our study, the sample size was relatively small. Another controversial issue might be that we conducted the heartbeat perception tasks after and not during the facial mirror-confrontations. Therefore, significant effects of the stimulation may have been missed. The task of heartbeat counting may have a distracting and relaxing effect by itself and may increase both IAc and cardiac vagal tone. Adding heartbeat detection tasks and also other ways to assess IAc, e.g., heartbeat discrimination tasks (Whitehead et al., 1977) during facial mirror-confrontations to the study protocol might shed more light on the acute self-focus effects on IAc in sDID patients. Moreover, we did not assess IAc after a neutral facial mirror-confrontation (without a negative or positive cognitive accompaniment). In addition, repeating the heartbeat detection task might have had a training effect, which might have confounded the results. The negative and positive conditions were not counterbalanced due to ethical reasons, i.e., we aimed at ending the experiment with a positive condition. However, there could have been a group receiving first a positive condition, then a negative condition and then again a positive condition which would not have been analyzed, but only inserted for ethical reasons. Furthermore, the relaxation task, which was performed before the baseline IAc assessment in order to minimize anticipatory anxiety, might have influenced the IAc baseline results. Heterogeneity in terms of comorbidities, psychotherapy experience and medication use might be considered another limitation of our study. This could not be ruled out since comorbidities are highly prevalent in patients suffering from sDID/DID (Freyberger and Spitzer, 2005). Our HCs sample might not be representative since all of them were hospital employees and medical students. Furthermore, we did not control for physical activity, a factor associated with improved IAc (Jones and Hollandsworth, 1981). Considering Kleckner et al. (2015) and Ring et al. (2015), it is necessary to take into account the methodological limitations of the Schandry task as well. Concerning the associations between cardiac vagal tone and IAc, our study design does not enable us to control for all possible confounders of heart rate variability in our sample, e.g., vigilance, emotional arousal, or physical activity. Analyzing heart rate variability dynamics and trait factors in general might have yielded interesting results in addition to the interindividual comparison.

Herein, we show for the first time substantially impaired IAc in sDID patients and first evidence that a higher cardiac vagal tone is correlated to better IAc in sDID patients. Consequently, it may be crucial to integrate therapy methods focusing on the perception of bodily signals into psychotherapy for sDID/DID patients, e.g., interoception training, body therapy or heart rate variability biofeedback. The body scan, an intervention from the Mindfulness Based Stress Reduction program (Fischer et al., 2017), and contemplative training (Bornemann and Singer, 2017), for instance, have proven to be potent interventions enhancing IAc in healthy participants.

## Conclusion

We observed a considerable IAc deficit at baseline in sDID patients compared to HCs. In sDID patients, higher cardiac vagal tone was associated with a more precise IAc. Our data might be considered to psychophysiologically support previous findings linking dissociative symptoms to compromised emotional experience, to impaired emotional learning and to a poor psychotherapy outcome. To the extent that bodily signal perception may play a causal role in sDID/DID, integrating therapeutic approaches improving IAc and cardiac vagal tone like IAc training, body therapy, contemplative training, or heart rate variability biofeedback into psychotherapy for sDID/DID patients might contribute to improving the psychotherapy outcome of this severely affected patient group.

## Author Contributions

ES and MS designed the study. ES conducted the experiments and wrote the first version of the manuscript. All of the authors contributed substantially to data analysis, interpretation of the results, and critical revisions of the manuscript. The final version was approved by all authors. All authors agreed to be accountable for all aspects of the work in ensuring that questions related to the accuracy or integrity of any part of the work are appropriately investigated and resolved.

## Conflict of Interest Statement

The authors declare that the research was conducted in the absence of any commercial or financial relationships that could be construed as a potential conflict of interest.
